# *miRNAsofAedesaegypti* (Linnaeus 1762) conserved in six orders of the class Insecta

**DOI:** 10.1038/s41598-021-90095-9

**Published:** 2021-05-21

**Authors:** Iram Pablo Rodríguez-Sanchez, Daniel Rafael Saldaña-Torres, Olga Karina Villanueva-Segura, Maria Lourdes Garza-Rodriguez, Mayra A. Gómez-Govea, Ghongwei Liang, María de Lourdes Ramírez-Ahuja, Margarita De La Luz Martinez-Fierro, Ivan Delgado-Enciso, Laura E. Martinez-de-Villarreal, Yu Zhou, Adriana E. Flores-Suarez, Xi Chen, Diana Resendez-Pérez, Chen-Yu Zhang, Gustavo Ponce-Garcia

**Affiliations:** 1 Universidad Autonoma de Nuevo Leon, Facultad de Ciencias Biologicas, Laboratorio de Fisiología Estructural y Molecular , Av. Universidad S/N CD. Universitaria, 66455 San Nicolas de los Garza, Nuevo Leon Mexico; 2 Universidad Autónoma de Nuevo León, Hospital Universitario “Dr. José Eleuterio González”, Centro Universitario Contra el Cáncer. , Monterrey, Nuevo Leon Mexico; 3 State Key Laboratory of Pharmaceutical Biotechnology, NJU Advanced Institute of Life Sciences, Jiangsu Engineering Research Center for MicroRNA Biology and Biotechnology, Nanjing University, Nanjing, 210093 China; 4 Laboratorio de Medicina Molecular, Universidad Autonoma de Zacatecas, Unidad Academica de Medicina Humana, 98160 Zacatecas, Zacatecas Mexico; 5 Facultad de Medicina, Universidad de Colima, 28040 Colima, Colima Mexico; 6 Instituto Estatal de Cancer, Secretaria de Salud de Colima, Colima, Mexico; 7 Universidad Autonoma de Nuevo Leon, Facultad de Medicina, Departamento de Genética , 64460 Monterrey, Nuevo Leon Mexico; 8 Universidad Autonoma de Nuevo Leon, Facultad de Ciencias Biologicas, Departamento de Zoologia de Invertebrados, Laboratorio de Entomologia Medica , Av. Universidad S/N CD. Universitaria, 66455 San Nicolas de los Garza, Nuevo Leon Mexico; 9 Universidad Autonoma de Nuevo Leon, Facultad de Ciencias Biologicas, Departamento de Biología Celular y Genética , 66455 San Nicolás de Los Garza, Nuevo Leon México

**Keywords:** Evolution, Genetics, Molecular biology, Physiology, Zoology

## Abstract

*Aedes* *aegypti* L. is the most important vector of arboviruses such as dengue, Zika, chikungunya, Mayaro, and yellow fever, which impact millions of people’s health per year. MicroRNA profile has been described in some mosquito species as being important for biological processes such as digestion of blood, oviposition, sexual differentiation, insecticide resistance, and pathogens dissemination. We identified the miRNAs of *Ae.* *aegypti* females, males and eggs of a reference insecticide susceptible strain New Orleans and compared them with those other insects to determine miRNA fingerprint by new-generation sequencing. The sequences were analyzed using data mining tools and categorization, followed by differential expression analysis and conservation with other insects. A total of 55 conserved miRNAs were identified, of which 34 were of holometabolous insects and 21 shared with hemimetabolous insects. Of these miRNAs, 32 had differential expression within the stages analyzed. Three predominant functions of miRNA were related to embryonic development regulation, metamorphosis, and basal functions. The findings of this research describe new information on *Ae.* *aegypti* physiology which could be useful for the development of new control strategies, particularly in mosquito development and metamorphosis processes.

## Introduction

Diptera is a major order that includes disease vectors of importance to human health. The mosquito *Aedes* *aegypti* (L.) belongs to the family Culicidae, one of the most dangerous to public health because the large number of diseases that it transmits to humans, such as dengue, Zika, yellow fever, chikungunya, and Mayaro viruses^1^. Disease dissemination occurs due to the effective invasive characteristics of *Ae.* *aegypti* such as the drying resistant eggs, and other independent physical factors such as climate change and urban sprawl^2^. Molecular physiology investigation of these organisms could lead to a better understanding in viral dissemination and adaptive ecology of vector insects.

MicroRNA (miRNA) is a small sequence of RNA (⁓ 22 nucleotides in length), which has genetic regulatory functions in eukaryotes^3^. These molecules contain within structure a region called seed, responsible for the hybridization specificity with a target transcript; the region size is 6 nucleotides (nt) and starts in the second nucleotide in the 5′ to 3′ direction^4^. *Ae.* *aegypti* (like many other organisms) has a physiologically fundamental relation with different miRNAs. Some miRNAs have been experimentally validated, including those that manage blood digestion and metamorphosis processes such as aae-miR-1890^5^, sexual dimorphism regulation such as aae-miR-309^6^, viral interactions such as aae-miR-375^7^ or bacterial diseases as in the case of aae-miR-12^8^ and aae-miR-2940-5p^9^. On the other hand, other miRNAs have been found silencing more than one target, allowing the complex physiological phenomena regulation for example, aae-miR-1174 which regulates diverse intestinal functions—sugar absorption, fluid excretion and blood intake^10^—or aee-miR-275 involve in blood digestion processes^11^.

Within the organisms of the class Insecta, miRNAs are molecules that have undergone constant alterations. This has revealed that miRNA conservation rates in insects are very low. An example of this miRNA conservation is that *Tribolium* *Castaneum* Herbst 1797 and *Drosophila* *melanogaster* Meigen 1830 have only a third of the total miRNAs conserved between them^12^.

Mosquitos conserve a large number of miRNAs such as miR-281, miR-184, miR-989, and miR-278, which are shared with *Ae.* *aegypti*, *Culex* *quinquefasciatus* Say 1832 and *Anopheles* *gambiae* Giles 1902^13^. Even so, there are miRNAs that are unique to every species as in the case of aae-miR-2946 for *Ae.* *aegypti* or cqu-miR-2951 and cqu-miR-2952 for *Cx.* *quinquefasciatus*^14^.

This is the first report of miRNA differential expression in comparison with other insects and three life stages of *Ae.* *aegypti*. The objective of this study was to describe the miRNA fingerprint of *Ae.* *aegypti* by Next Generation Sequencing. This information is especially relevant as it can be a powerful tool for the proposal of new strategies for targeted species control without affecting ecologically important organisms.

## Results

### miRNAs annotation

A total of 21,632,581 reads were obtained; 40,815 showed similarity in the structure and biogenesis of 80 different miRNAs of *Aedes* *aegypti.* The number of readings for each biological stage is provided in Table 1.

**Table 1:** miRNA annotation by biological stage.

Stage	Total reads	Mapped reads	miRNAs read with a miRDeep2 (score ≥ 4) reported for *Aedes* *aegypti*
Eggs	4,474,188	1,691,089 (~ 38%)	16,198 (66*)
Females	9,409,689	4,613,825 (~ 49%)	14,490 (72*)
Males	7,748,704	4,017,274 (~ 52%)	10,127 (61*)
Total	21,632,581	10,322,188 (~ 48%)	40,815 (80*)

% Correspond to the number of reads recovered with respect to their total number in each of the approaches.

*Correspond to the number of miRNAs previously reported to *Ae.* *aegypti* in miRDeep2.

### miRNAs taxonomic distribution

**Figure 1:**
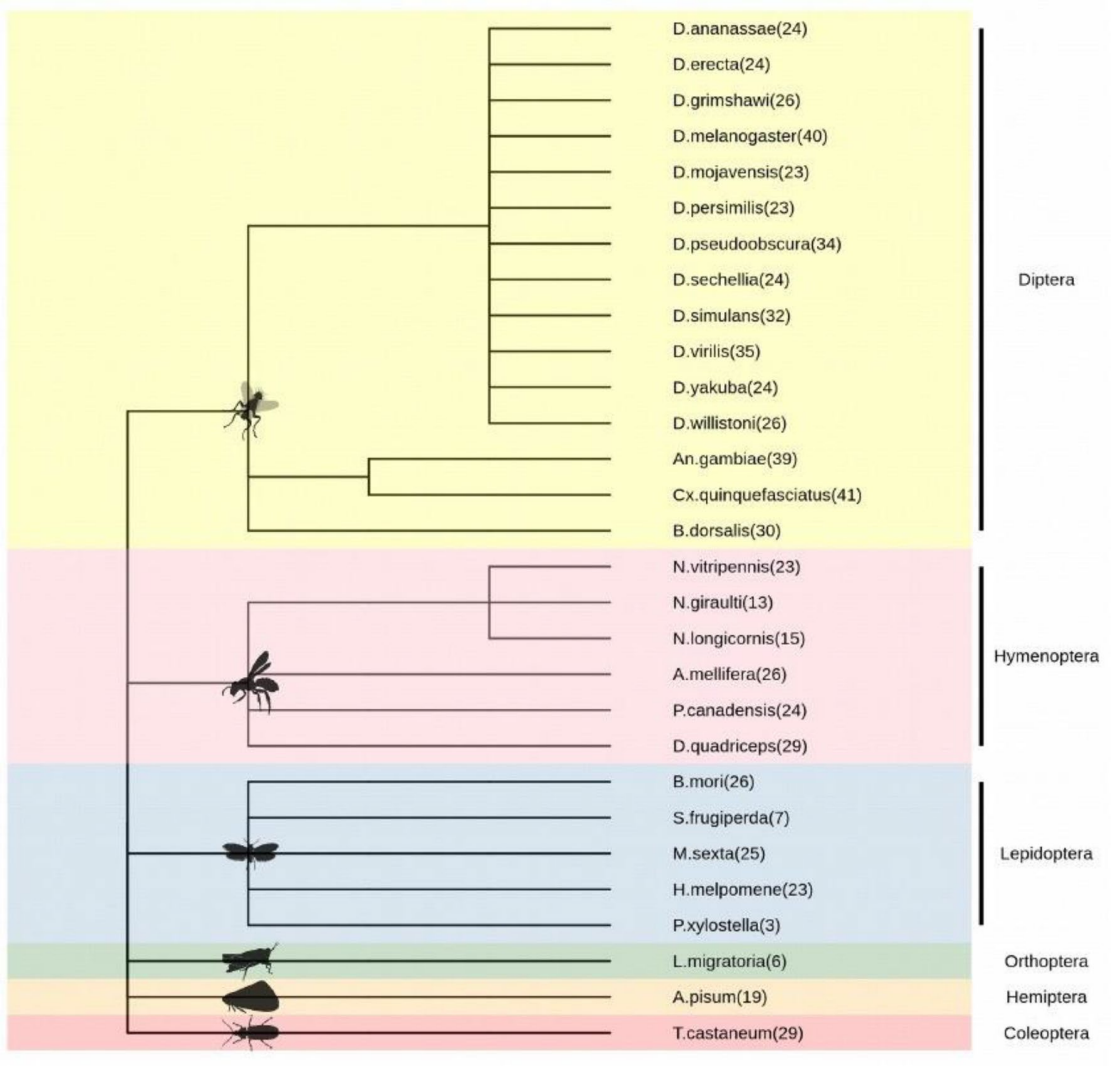
Taxonomic distribution of the 713 conserved miRNAs in class Insecta and their abundance by species.

Of the 80 miRNAs annotated from *Ae.* *aegypti,* 55 showed significant conservation with 713 miRNAs from other insect species, while 25 showed *Ae.* *aegypti-*specific conservation, considering as cut-off an E value ≤ 0.005. According to the NCBI taxonomy information of these insect species, we identified that of the 713 miRNAs, 445 miRNAs (62.41%) were present in the order Diptera, 130 in Hymenoptera (18.23%), 84 in Lepidoptera (11.78%), 29 in Coleoptera (4.07%), 19 in Hemiptera (2.66%) and 6 in Orthoptera (0.84%) as described in Fig. 1 and Supplementary Material 1.

### Tendency of *Ae.aegypti* miRNAs conservation in taxonomic orders

**Figure 2:**
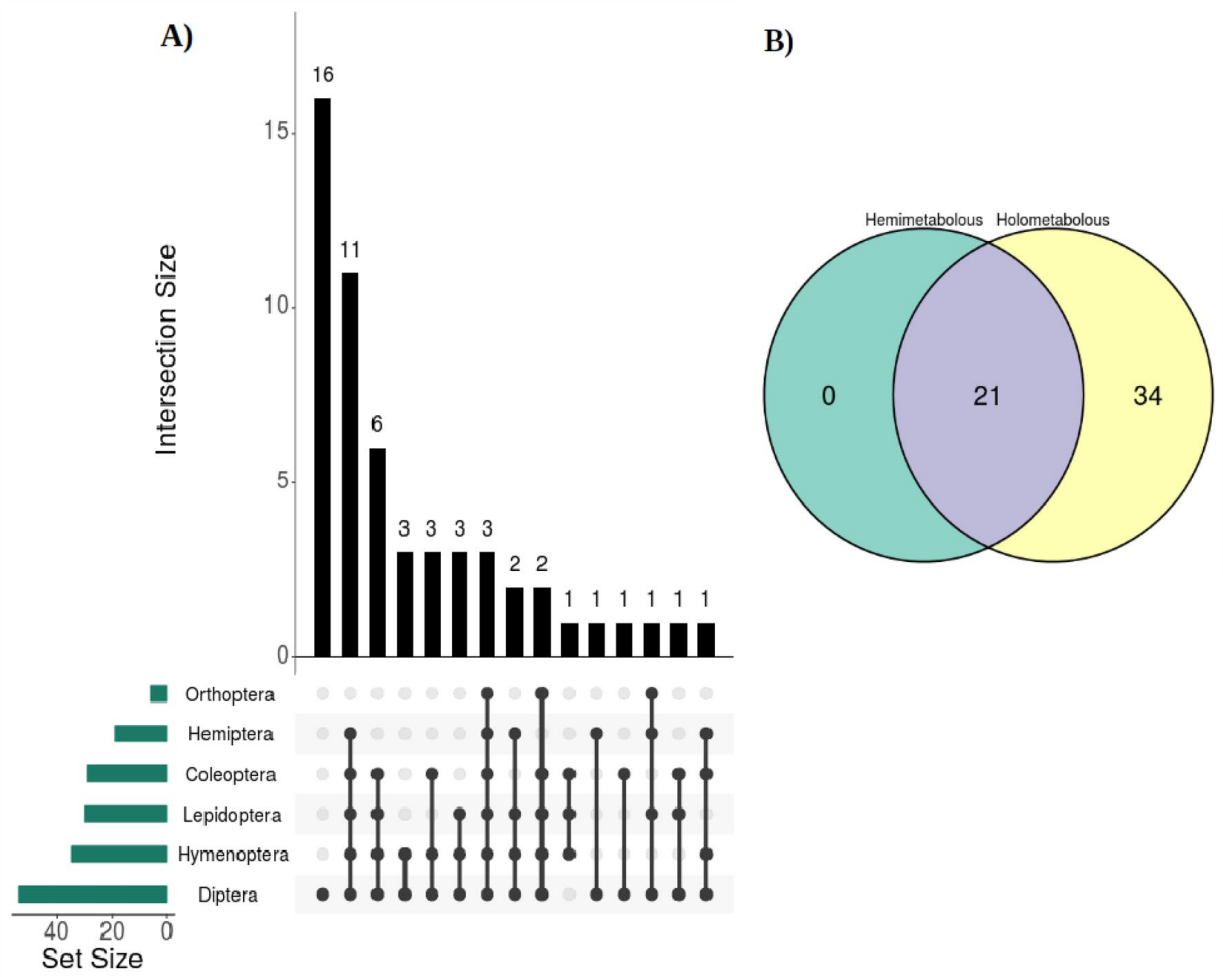
An interaction graph of the 55 conserved miRNAs from *Ae.* *aegypti* in the different orders analyzed. (**A**) In the UpSet diagram, on the x-axis, a 15 different conservation pattern matrix with miRNAs representing the 6 orders (dotted black lines) is presented; likewise, miRNA abundances of each pattern are illustrated on the y-axis in black bar graph form. (**B**) Distribution Venn diagram of the 55 miRNAs between the two metamorphoses types analyzed.

Of the 55 conserved miRNAs, 15 conservation patterns were obtained in the six orders previously mentioned (x-axis, Fig. 2A). We found specifically for *Diptera* 16 miRNAs conserved (y-axis in bar graph form, Fig. 2A). Also, of the 55 conserved miRNAs, 34 were only found in holometabolous insect orders (Diptera, Lepidoptera, Coleoptera and Hymenoptera) and 21 miRNAs were shared with hemimetabolous orders—Hemiptera and Orthoptera (Fig. 2B); none of miRNAs was unique to hemimetabolous orders.

### miRNA presence in females, males and eggs

**Figure 3:**
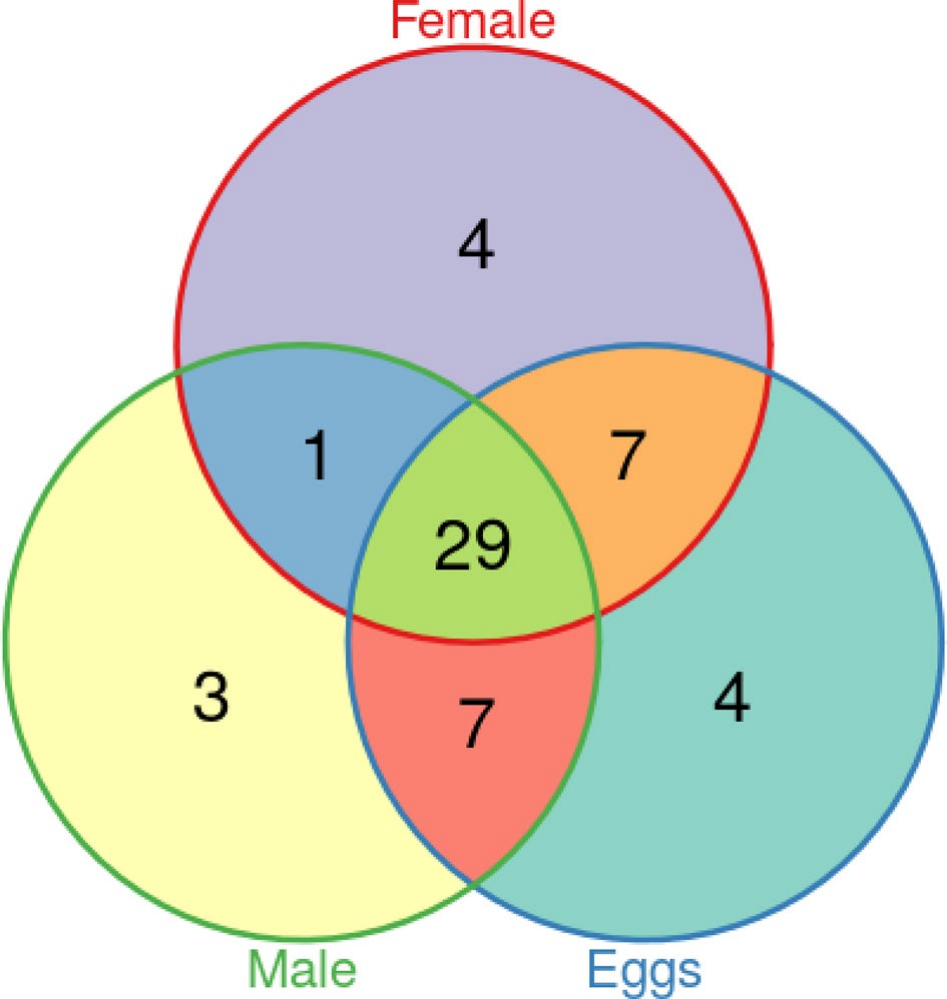
Venn diagram with the distribution of miRNAs conserved in organisms of the class Insecta among the three life stages analyzed.

Figure 3 showed 55 conserved miRNAs: 4 showed the specific distribution in females (miR-1175-5p, miR-193, miR-285 and miR-31), 3 in males (miR-263a-5p, miR-71-5p and miR-993) and 4 in eggs (miR-2944a-5p, miR-307, miR-996 and miR-33). Of the 44 remaining miRNAs, 7 were found in females and eggs, 7 in males and eggs, 1 in females and males, and 29 miRNAs were shared among females, males and eggs (Supplementary Material 2).

### Differential expression of miRNAs in three life stages and conserved in the class Insecta

**Figure 4:**
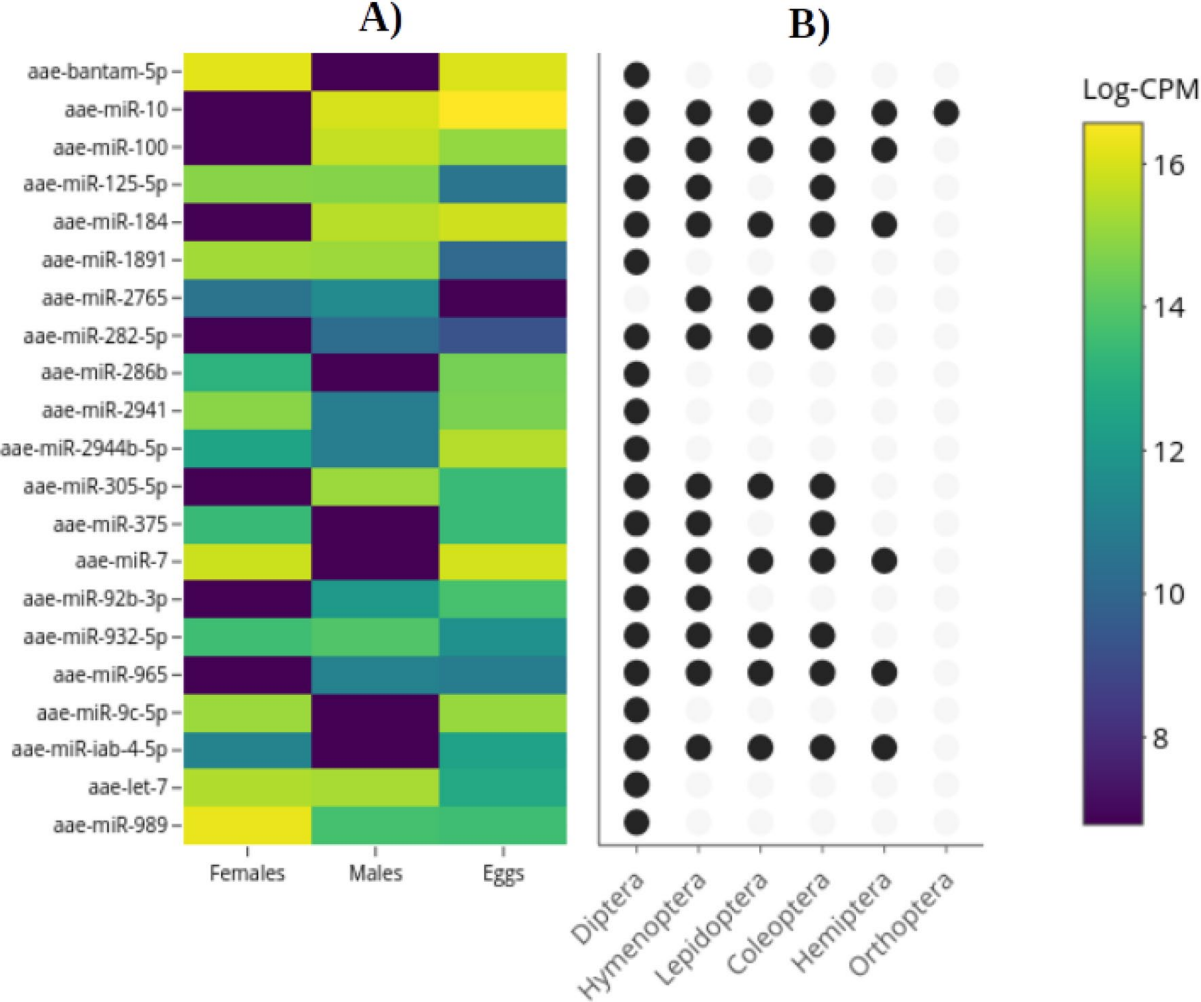
Differential expression of 21 miRNAs in female, male and eggs from *Ae.* *aegypti* and their distribution in the class Insecta. (**A**) Heatmap of the 21 miRNAs with DE (y-axis) in 3 approaches analyzed (x-axis) and (**B**) conservation patterns of each miRNA between insect orders.

LRT was used to analyze the 44 miRNAs that showed expression in more than one life stage; among these, we found that only 21 miRNAs exhibited differential expression (DE) in the three contrasts (Fig. 4A). These were considered downregulated or upregulated, depending on their statistical significance (LR > 4 and p value ≤ 0.01) and fold change behavior (negative or positive) in comparison to their mean expression among the three life stages (Supplementary Material 2). A total of 17 miRNAs were downregulated; of these, five were in males (bantam-5p, miR-286b, miR-375, miR-7, miR-9c-5p), seven in females (miR-10, miR-100, miR-184, miR-282-5p, miR-305-5p, miR-92b-3p, miR-965) and five in eggs (miR-125-5p, miR-1891, miR-2765, miR-932-5p, let-7). We also identified four upregulated miRNAs, one in females (miR-989) and two in eggs (2944b-5p, miR-iab-4-5p); one miRNA was present in both females and eggs (miR-2941).

Of the 21 miRNAs with DE found in females, males and eggs of *Ae.* *aegypti* have been found in six orders of Insecta as shown in Fig. 4B: in Diptera, eight miRNAs were specific (bantam-5p, miR-1891, miR-286b, miR-2941, miR-2944-5p, miR-9c-5p, let-7, miR-989). In all the holometabolous, three miRNAs were conserved (miR-282-5p, miR-305-5p, miR-932-5p) and four miRNAs were present in some holometabolous (miR-125-5p, miR-2765, miR-375, miR-92b-3p), a particular case, miR-2765 was present in the orders Coleoptera, Lepidoptera, and Hymenoptera but not Diptera; five miRNAs were shared between holometabolous and the order Hemiptera (miR-100, miR-184, miR-7, miR-965, miR-iab-4-5p). Finally, the miRNA conserved in all holometabolous and hemimetabolous orders was miR-10.

### miRNAs biological function in *Ae.aegypti*

**Figure 5:**
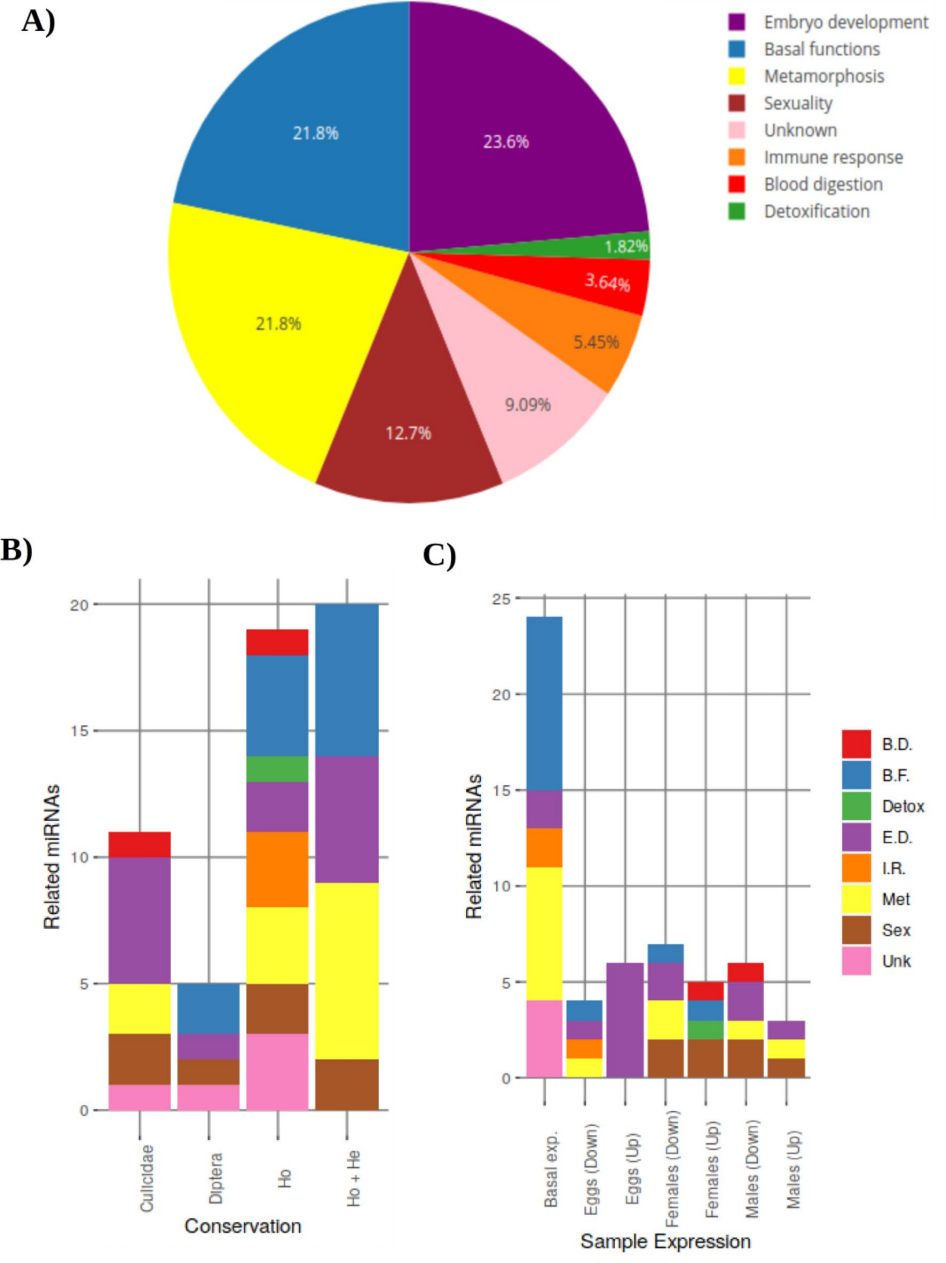
Biological function correlation with abundance of the 55 miRNAs in *Ae.* *aegypti.* (**A**) miRNAs distribution correlated with biological function, (**B**) miRNA distribution based on categorization clusters and (**C**) miRNAs expression levels in three life stages. *Ho* holometabolous, *He* hemimetabolous.

We found 55 miRNAs from *Ae.* *aegypti* using miRDeep2 (biological database) tool (Supplementary Material Table S1). Figure 5A shows that these 55 were distributed in 8 different biological function categories, which in percentage were as follows: embryo development (ED) 23.6%, basal functions (BF) 21.8%, metamorphosis (Met) 21.8%, sexuality (Sex) 12.7%, unknown (Unk) 9.09%, immune response (IR) 5.45%, blood digestion (BD) 3.64% and detoxification (Detox) 1.82%.

There were 4 categorization clusters (Fig. 5B): Culicidae with high DE proportion, Diptera with BF, holometabolous (Ho) with BF and holometabolous + hemimetabolous (Ho + He) with Met. Three parameters had expression levels (upregulated, basal and downregulated expression) and life stages (eggs, females and males) (Fig. 5C), where eggs showed DD upregulation and BD, BF, IR and Met downregulation; females showed BD, BF, Detox and Sex upregulated. Finally, males exhibited BD downregulated.

## Discussion

Metamorphosis diversity in insects (ametabolous, hemimetabolous and holometabolous) is a fundamental part of their evolution, being a determining factor in the conservation of these organisms^15^. So far, different miRNAs, such as miR-let-7, miR-100 and miR-125, have been conserved and participate in metamorphosis in different organisms such as *Blattella* *germanica* Linnaeus, 1767 (hemimetabolous)^16^ and *Anopheles* *gambiae* (holometabolous)^17–19^. We showed that 34 miRNAs corresponded to only one or more holometabolous organism, classified in the orders Diptera, Hymenoptera, Lepidoptera and Coleoptera and that 21 miRNAs shared conservation with some hemimetabolous insects.

We observed an abundance of miRNAs, i.e., 12, related to the metamorphosis process (Fig. 5A, yellow segment, and Supplementary Table S1); these miRNAs have been associated with different metamorphosis functions in other insects, such as bantam-5p^11^, let-7, miR-252-5p^20^ and miR-315-5p^21^, regulating wing development. miR-263a-5p^22^ and miR-282-5p^23^ have been found in neuronal regulation signals. miR-1890 regulates JHA15 juvenile hormone expression in *Ae.* *aegypti*^5^*;* miR-133 is upregulated in *Drosophila* *melanogaster* pupation^24^. Of the 12 miRNAs related to the metamorphosis process, eight (let-7, miR-14, miR-263a-5p, miR-276-3p, miR-276-5p, miR-315-5p and miR-965) have been found to be conserved in holometabolous as well as hemimetabolous organisms (Fig. 5B, Ho + He). Some of these miRNAs have been reported to be in great abundance in insects^25–27^ and have been associated with wing development in hemimetabolous metamorphosis in *B.* *germanica*^28–30^. This may suggest that the development of wings has been regulated at the miRNomic level by organisms conserved along the evolution of the Insecta.

Another portion contains 13 miRNAs (Fig. 5A, purple section; Supplementary Table S1); six of these showed overexpression in eggs [Fig. 5C, second bar “Eggs (Up)”, (Supplementary Table S1)], but they are not related to specific biological events as yet. We observed miR-10 and miR-71-5p overexpression in holometabolous and hemimetabolous insects. Only five have been described previously in *D.* *melanogaster* embryos playing developmental functions: miR-307 regulated cell death processes through the silencing of *wntless*^6^; miR-33 in cell proliferation events by silencing the genes *CDK6* y *CCND1*^31^; miR-9a in muscle myofibril development by silencing troponin-T^32^; and miR-9c-5p in the cleaning of unstable maternal transcripts^33^. Finally, miR-iab-4-5p regulating the distribution and development of the wings and halters through the silencing of Ultrabithorax^34^. Thus, we can suggest embryonic development function for these 5 miRNAs in *Ae.* *aegypti*.

Five miRNAs (miR-1891, miR-286b, miR-2944a-5p, miR-2944b-5p and miR-996) were found conserved only in the members of the family Culicidae (Fig. 5B, bar 1 “Culicidae”, Supplementary Table S1); only two (miR-1891 and miR-996) have been reported overexpressed in eggs of *Ae.* *aegypti*, *Anopheles* *gambiae* and *An.* *stephensi* Liston 1901^35,36^, and three (miR-286b, miR-2944a-5p, miR-2944b-5p) were highly expressed in the early development stages of mosquitoes^35,37^. The family formed by miR-996, miR-279, and miR-296 have undergone some duplication events along insect evolution, making it diversify in holometabolous organisms^38^. Thus, we suggested that miRNAs conserved specifically in Culicidae could have undergone similar events that would have led to miRNomic regulation in embryonic stages in a specific way.

Discrepancies were found between previous studies and our findings regarding the following miRNAs: miR-9a has been reported in embryo development function in *D.* *melanogaster* eggs^32^, but in contrast, no significant changes were found in the biological approaches that we used (eggs, females and males); miR-71-5p with overexpression in *Manduca* *sexta* (Linnaeus 1763) eggs^39^ was in contrast to the expression in males in our findings; miR-10 was overexpressed in *D.* *melanogaster*, *T.* *castaneum*, *Apis* *mellifera* (Linnaeus 1758) and *B.* *germanica* eggs^40,41^ and downregulated in females according to our results. Thus, as explained above, we can infer new and different roles for these miRNAs in *Ae.* *aegypti*.

Twelve miRNAs conserved between *Ae.* *aegypti* and the class Insecta were categorized in diverse basal functions such as neuronal signaling, hypoxia functions, nervous system, and circadian cycle (Fig. 5A, blue section; Supplementary Table S1). The functions of these miRNAs are: miR-1, regulating the architecture and maintenance of *D.* *melanogaster* cardiac tissue through DELTA silencing^21^; miR-137, in neuronal signaling of *Drosophila* Parkinson disease models^42^; miR-190, orchestrating the functions related to hypoxia in *D.* *melanogaster*^43^; miR-263b-5p and miR-279, in the circadian cycle in *D.* *melanogaster*^44^; miR-2c and miR-31, in the maintenance of the nervous system of *D.* *melanogaster*^45,46^ and miR-957, in the interaction *Cx.* *quinquefasciatus*/Western Nile virus (WNV)^47^; miR-9b, silencing serine/threonine kinase protein in *Drosophila*^48^; miR-932-5p, in the neuroplasticity mechanisms and long-term memory of *A.* *mellifera*^49^; miR-305-5p, in the intestinal homeostasis of *D.* *melanogaster*^50^; and miR-8-5p, in post-embryonic development^51^ and growth factor regulation of body fat in larval stages^40^ in *D.* *melanogaster*.

Of these 12 miRNAs, 9 showed a basal expression in the life stages of *Ae.* *aegypti* analyzed in the present study (Fig. 5C, bar 1 “Basal exp.”; Supplementary Table S1) and the remaining 3, were downregulated in eggs (miR-932-5p) or upregulated (miR-31) and downregulated (miR-305-5p) in females. Conservation profiles of the 12 miRNAs showed 4 with conservation only in holometabolous, 6 in holometabolous and hemimetabolous and 2 specifically in Diptera (Fig. 5B). Of the basal function of the miRNAs with holometabolous and hemimetabolous conservation, miR-2c and miR-8-5p have different biological functions depending on the type of metamorphosis that was explored. For the hemimetabolous functions for these miRNAs, miR-2c was related to metamorphosis processes through the silencing of *KR-H1* in *B.* *germanica*^28^ and miR-8-5p in chitin biosynthesis in *Locusta* *migratoria* Linnaeus 1758^52^ and motor coordination of *B.* *germanica*^53^.

Other biological functions were observed (sex, blood digestion and detoxification) that despite having a low abundance are relevant in the biology of *Ae.* *aegypti*. miRNAs were found to be related to sexual development, where 2 were found to be unique for Culicidae (miR-2942 and miR-989) (Fig. 5B, bar 1 “Culicidae”; Supplementary Table S1), which regulates ovary development in *Ae.* *aegypti*, *Cx.* *quinquefasciatus* and *An.* *stephensi*^14,54–56^.

On the other hand, miR-193 was shown to be conserved in the order Diptera (Fig. 5B, bar 2 “Diptera”; Supplementary Table S1), being overexpressed in *D.* *melanogaster*^57^. Also, the experimental design did not consider sex as a factor for expression analysis, so our results pointed to be a possible function in feminization. The battery of miR-7 and miR-993 was conserved in the holometabolous organisms (Fig. 5B, bar 3 “Ho”; Supplementary Table S1), where it has been observed that they fulfill masculinization roles in *D.* *melanogaster*^58,59^. Another battery of miR-184 and miR-100 was conserved in holo- and hemimetabolous insects (Fig. 5B, bar 4 “Ho + He”; Supplementary Table S1), both being highly expressed in males of *An.* *anthropophagus* Xu & Feng 1975^60^. miR-184 have been associated with regulation of ovary development in *D.* *melanogaster*^21^ and with high expression in females of the 6^th^ nymphal stage in *B.* *germanica*^30^ and miR-100 with essential metamorphosis regulation conserved among holometabolous and hemimetabolous insects^28^. Our results suggest that the feminization of mosquitoes may require a miRNomic regulation conserved uniquely in Culicidae.

Two miRNAs were found associated with blood digestion (Fig. 5A, red section); the miR-1175-5p was strictly conserved in Culicidae (Fig. 5B, bar 1 “Culicidae”; Supplementary Table S1) being in high concentrations in females of *An.* *stephensi*, *An.* *gambiae,* and *Cx.* *quinquefasciatus*^14,36^, and miR-375 regulating the silencing of immunological cactus genes and REL1 in *Ae.* *aegypti*^7^.

Finally, only miR-285 was found to be associated with detoxification functions. Previously, it has been described in a resistant strain (91-R) of *D.* *melanogaster* in exposure to DDT^61^. This agrees with our results of overexpression in females because resistant strains were not analyzed in this study, and we used the susceptible reference strain New Orleans. However, expression findings infer that miR-285 plays an essential role in the development of insecticide resistance in a specific way in Diptera.

In conclusion, the sequencing of miRNAs in eggs and adults (males and females) of *Ae.* *aegypti* showed a total of 55 miRNAs with relevant conservation with other members of the class Insecta. Of these miRNAs, 32 showed DE in the stages analyzed, showing to be related to development, metamorphosis, and sex functions, among others. On the other hand, 12 miRNAs with basal expressions were shown to be mainly related to diverse biological functions. While a lower abundance of conservation was expected in specific functions, such as detoxification, blood digestion, and immune response, there was a predominance of highly conserved miRNAs related to embryonic development and metamorphosis processes or a diversity of conservation clusters in miRNAs related to sexuality. These results suggest that the development of wings, with miR-iab-4-5p and masculinization, with the battery of miR-7 and miR-993, could be ancestral miRNomic mechanisms.

We propose to coin the word "phylorthology" to refer to the phylogenetic relationship that species have with relation in a conserved sequence in 100%.

## Methods

### General strategy

The general strategy consisted of obtaining miRNA sequences from 3 life stages in *Ae.* *aegypti* (males, females, and eggs). Total RNA was extracted, and the corresponding miRNA fractions were sequenced. These sequences were annotated and subjected to conservation analysis against the miRNAs available in the class Insecta. Those with significant conservation (E value ≤ 0.005) were subjected to interaction graphics and cladograms, to observe their abundance and diverse patterns of conservation between the organisms of the class Insecta. Finally, a DE analysis of these miRNAs was evaluated using a likelihood ratio statistical analysis.

### Sample collection

*Ae.* *aegypti* specimens belonging to the New Orleans strain were bred at the insectarium of the Medical Entomology Laboratory of the Facultad de Ciencias Biologicas in the Universidad Autonoma de Nuevo Leon. Breeding of the mosquitoes was performed under laboratory conditions with a temperature of 28 ± 2 °C, a light/dark photoperiod of 12/12 h and relative humidity of 70 ± 2%. A total of 3000 eggs, 100 males and 100 females were separated, and miRNA extraction from the specimens was carried out with the commercial kit miRNeasy Mini, following the provider instructions (Qiagen, Germantown, MD, USA). RNA extracted was treated with RQ1 RNase-Free Dnase (Promega, San Luis Obispo, CA, USA) to remove genomic DNA traces. Standard spectrophotometry (Thermo Fisher, Waltham, MA, USA) and agarose gel electrophoresis were used to evaluate RNA purity and integrity, respectively.

### Next generation sequencing

RNA of acceptable quality and quantity was sent to BGI Global Genomics Services (Yantian District, Shenzhen, China) for sequencing of the small fraction (less than 30 base pairs) with Illumina Solexa technology and ligation of pair adaptors to their 5′ and 3′ ends.

### miRNA annotation

miRNA annotations were made with the tool miRDeep2^62^, using as a reference genome the assembled version of *Ae.* *aegypti* (AaegL5), and as a precursor reference the available structures of miRNAs in miRBase v22^63^ of the 30 organisms of the subphylum Hexapoda. The analysis was run with a minimum cut-off of 18 nt, and only the miRNAs that showed a miRDeep score ≥ 4 were considered, on the basis of those described by Friedländer et al.^62^ and our own criteria.

### Phylogenetic and conservation analysis

The annotated miRNAs that had phylogenetic conservations (phyloortology) with organisms of class Insecta were identified through local alignments against the database of miRNAs available for Hexapoda with the BLASTN tool of miRBase. Mature and precursor sequences with E values ≤ 0.005 were considered conserved and those with values > 0.005 were discarded. Subsequently, the abundance of miRNAs conserved with our results were quantified and categorized by insect species. A cladogram was generated with taxonomic data^64^ of every species with the tools of the software Rv3.6.0^65^: ggtree1.14.6^66^, treeio1.6.2^67^, devtools2.0.2^68^, phytools0.6–72^69^ and ggimage 0.2.1^70^. miRNA presence in the different insect orders and metamorphosis types (holometabolous and hemimetabolous) were observed. These trend analyses were made through visualizations of interaction of UpSet and Venn diagrams through the use of the graphic interactive tool intervene^71^ and the package UpSetRv1.3.3^72^.

### Differential expression analysis

For the expression analyses, only miRNAs that showed conservation were considered; the total reads were obtained by the mean of each of the approaches explored. A Venn diagram was made to show the dispersion of these miRNAs between the 3 different biological approaches (eggs, females and males) and to discard the miRNAs with specific expression in only one explored biological approach. The mean of the readings was put through depuration and normalization protocols according to Law et al.^73^, where data expression of the readings was transformed into counts per million (CPM). This value was expressed in log2 (log2-CPM) and was normalized using Trimmed Mean of M values (TMM) method. For the determination of the miRNAs with DE in the three contrasted approaches (eggs vs females, eggs vs males and, females vs males), a generalized linear model and a likelihood ratio likelihood (LRT) were used^74^. The miRNAs with a DE were considered if they showed LRT values of > 4 and a p value ≤ 0.01; their expression values (log2-CPM) were used for the plotting of graphs and heat maps through the R tools of graphics development heatmaply^75^, Glimma^76^ and RColorBrewer^77^. The R tools limma 3.40.0^78^ and edgeR 3.24.3^79^ were used for statistical analyses.

## Supplementary Information

Supplementary Information 1.

Supplementary Information 2.

Supplementary Information 3.
